# Supplementary material for: Effect of automated versus conventional ventilation on mechanical power of ventilation—A randomized crossover clinical trial
Source: PLoS One. 2024 Jul 30;19(7):e0307155. doi: 10.1371/journal.pone.0307155 (PMC11288413; doi:10.1371/journal.pone.0307155)
Supplement: S1 File — (PDF) [file pone.0307155.s002.pdf]

RESEARCH PROTOCOL

The Effect of Closed–loop versus Conventional Ventilation on Mechanical Power (INTELLiPOWER) – study protocol for a multicenter crossover randomized clinical trial

Laura A. Buiteman–Kruizinga[1,3], Michela Botta3, Anissa Tsonas[3], Lotte Spies[3], Hassan E. Mkadmi[2], Stephanie List[5], Mariska Brink[5], Ben de Boer[5], Patricia van Velzen[5], Ary Serpa Neto[3,6,7], Marcus J. Schultz[3,8,9], Pim L.J. van der Heiden[1] and Frederique Paulus[3,10]

Reinier de Graaf Hospital, Delft, the Netherlands
[1]Department of Intensive Care
[2]Department of Research

Academic Medical Center, Amsterdam, the Netherlands:
[3]Department of Intensive Care
[4]Laboratory of Experimental Intensive Care and Anesthesiology (L·E·I·C·A)

Dijklander Hospital, Hoorn, the Netherlands:
[5]Department of Intensive Care

Monash University, Melbourne, Australia:
[6]Australian and New Zealand Intensive Care-Research Centre (ANZIC-RC)

Hospital Israelita Albert Einstein, São Paulo, Brazil:
[7]Department of Critical Care Medicine

Mahidol University, Bangkok, Thailand:
[8]Mahidol–Oxford Tropical Medicine Research Unit (MORU)

University of Oxford, Oxford, UK:
[9]Nuffield Department of Medicine

Amsterdam University of Applied Sciences, Faculty of Health, Amsterdam, The Netherlands:
[10]ACHIEVE, Centre of Applied Research

Correspondence:
Laura A. Buiteman-Kruizinga
Reinier de Graaf Hospital
Reinier de Graafweg 5
2625 AD Delft
Email: l.kruizinga@rdgg.nl

PROTOCOL TITLE: The Effect of Closed–loop versus Conventional Ventilation on Mechanical Power (INTELLiPOWER) – study protocol for a multicenter crossover randomized clinical trial

**Protocol ID**	INTELLiPOWER
**Short title**	The effect of Closed–loop Ventilation on Mechanical Power
**Version**	3.0
**Date**	June 11, 2021
**Coordinating investigator/project leader**	Laura A. Buiteman-Kruizinga<br>Reinier de Graaf Hospital<br>Reinier de Graafweg 5<br>2625 AD Delft<br>Telephone: +31 15 2604040<br>Email: l.kruizinga@rdgg.nl<br>&<br>Frederique Paulus, PhD<br>Department of Intensive Care<br>Amsterdam University Medical Centers, location 'AMC'<br>Meibergdreef 9<br>1105 AZ Amsterdam, The Netherlands<br>Telephone: +31 20 5669111<br>E-mail: f.paulus@amsterdamumc.nl
**Principal investigator(s) (in Dutch: hoofdonderzoeker/ uitvoerder)**	Prof. M.J. Schultz, MD PhD<br>Department of Intensive Care<br>Amsterdam University Medical Centers, location 'AMC'<br>Meibergdreef 9<br>1105 AZ Amsterdam, The Netherlands<br>Telephone: +31 20 5662509<br>E-mail: marcus.j.schultz@gmail.com
**Sponsor (in Dutch: verrichter/opdrachtgever)**	Amsterdam University Medical Centers, location 'AMC'<br>Meibergdreef 9<br>1105 AZ Amsterdam, The Netherlands
**Independent expert(s)**	J. Horn, MD PhD<br>Department of Intensive Care<br>Amsterdam University Medical Centers, location 'AMC'<br>Meibergdreef 9<br>1105 AZ Amsterdam, The Netherlands<br>Telephone: +31 20 5669111<br>E-mail: j.horn@amsterdamumc.nl

PROTOCOL SIGNATURE SHEET

Name	Signature	Date
**Head of Department:**<br><br>Prof. M.B. Vroom, MD PhD	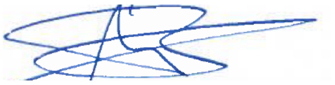	20 maart 2022
**Principal Investigator:**<br><br>Prof. M.J. Schultz, MD PhD	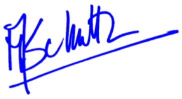	20 maart 2022

TABLE OF CONTENTS

1. INTRODUCTION AND RATIONALE ... 8
1.1 Invasive ventilation ... 8
1.2 Mechanical Power of ventilation ... 8
1.3 Fully–automated or closed–loop ventilation ... 8
1.4 Need for this study ... 9
2. OBJECTIVE AND HYPOTHESIS ... 10
2.1 Objective ... 10
2.2 Hypothesis ... 10
3. STUDY DESIGN ... 11
4. STUDY POPULATION ... 12
4.1 Population ... 12
4.2 Inclusion criteria ... 12
4.3 Exclusion criteria ... 12
4.4 Sample size calculation ... 12
5. METHODS ... 13
5.1 Primary endpoint ... 13
5.2 Secondary endpoints ... 13
5.3 Randomization, blinding and treatment allocation ... 13
5.4 Study procedures ... 13
5.5 Data collection ... 14
5.5.1 Demographic and general data ... 14
5.5.2 Baseline ventilator settings, variables and parameters ... 14
5.5.3 Study parameters ... 15
5.5.4 Blood gas data ... 16
5.5.5 Follow–up data at day 7 and day 28 ... 16
5.6 Automatic data collection ... 16
5.7 Withdrawal of individual subjects ... 16
5.8Replacement of individual subjects after withdrawal ... 16
5.9 Follow-up of subjects withdrawn from treatment ... 16
5.10 Premature termination of the study ... 16
6. SAFETY REPORTING ... 17
6.1 Temporary halt for reasons of subject safety ... 17

6.2 Serious adverse events (SAE) 17
6.3 Follow-up of adverse events..... 17
6.4 Data Safety Monitoring Board (DSMB)..... 17
7. STATISTICAL ANALYSIS..... 18
8. ETHICAL CONSIDERATIONS..... 19
8.1 Regulation statement 19
8.2 Recruitment and consent..... 19
8.3 Benefits and risks assessment, group relatedness 19
8.4 Compensation for injury 19
9. ADMINISTRATIVE ASPECTS, MONITORING AND PUBLICATION 20
9.1 Handling and storage of data and documents..... 20
9.2 Monitoring and Quality Assurance..... 20
9.3 Amendments 20
9.4 Annual progress report..... 20
9.5 Temporary halt and (prematurely) end of study report 20
9.6 Public disclosure and publication policy 20
10. REFERENCES..... 21
APPENDIX I 23
APPENDIX II 24

ABBREVATIONS AND DEFINITIONS

APACHE	Acute Physiology and Chronic Health Evaluation
$EtCO_2$	End-tidal Carbon Dioxide
FiO_2	Fraction of inspired Oxygen
ICU	Intensive Care Unit
I:E ratio	Inspiratory to Expiratory ratio
MP	Mechanical Power
PEEP	Positive End–Expiratory Pressure
Pplateau	Plateau pressure
Pmax	Maximum airway pressure
Ppeak	Peak pressure
RR	Respiratory Rate
RR_{set}	Set Respiratory Rate
SaO_2	Saturation of Arterial Oxygen
SpO_2	Saturation of peripheral Oxygen
V_T	Tidal Volume
VILI	Ventilator induced lung injury
ΔP	Driving pressure

SUMMARY

Rationale

Mechanical ventilation can cause ventilator–induced lung injury (VILI). Lung protective ventilation, consisting of a low tidal volume (V_T), a low plateau pressure (Pplateau) and a low driving pressure (ΔP) improves survival and shortens duration of ventilation in patients with acute respiratory distress syndrome (ARDS). Lung protective ventilation may also benefit critically ill patients with respiratory failure not caused by ARDS. 'Mechanical Power of ventilation' (MP), the amount of energy per time transferred from the ventilator to the respiratory system, is a summary variable that includes all the components that play a role in VILI. With fully–automated closed–loop ventilation, these components are no longer set by the operator, but under control of the algorithms in the ventilator.

Objective

To compare MP under INTELLiVENT–adaptive support ventilation (ASV), a fully–automated closed–loop ventilation, with MP under conventional ventilation.

Hypothesis

INTELLiVENT–ASV compared to conventional ventilation results in a lower MP.

Study design

National, multicenter, crossover, randomized clinical trial.

Study population

Invasively ventilated critically ill patients.

Methods

The ventilator will be randomly switched between INTELLiVENT–ASV for 3 hours and conventional ventilation for 3 hours. The amount of MP is calculated using various equations proposed in the literature.

Study endpoints

The primary endpoint is the amount of MP with each form of invasive ventilation.

Nature and extent of the burden and risks associated with participation, benefit and group relatedness

Differences in burden and risks of the two ventilation strategies compared in the current study are not expected. Both modes of ventilation are interchangeably used as part of standard care in the participating centers. No other interventions are performed. Neither the collection of demographic and outcome data, nor the capturing of ventilation characteristics causes harm to patients.

1. INTRODUCTION AND RATIONALE

1.1 Invasive ventilation

Invasive ventilation is an often needed and at times even life–saving intervention, but has the potential to cause so–called ‘ventilator–induced lung injury’ (VILI). VILI is at least in part due to overdistension of the lungs, and is associated with a higher mortality and longer duration of mechanical ventilation [1]. ‘Lung–protective’ ventilation consisting of a low tidal volume (V_T), a low plateau (Pplateau) [2] and a low driving pressure (ΔP) [3] improves survival and is leads to a faster liberation from the ventilator in patients with acute respiratory distress syndrome (ARDS) [4]. Lung–protective ventilation may also benefit critically ill invasively ventilated patients not having ARDS.

1.2 Mechanical Power of ventilation

The ‘Mechanical Power of ventilation’ (MP) is the amount of energy per time transferred from the ventilator to the respiratory system. While this energy is mainly used to overcome airway resistance (R_{aw}) and respiratory system compliance (C_{RS}), part of it can act directly on lung tissue, causing VILI [5,6]. MP has been shown to be associated with important patient–centered outcomes in critically ill patients in need of invasive ventilation [7]. MP is a summary variable that includes all components that play a role in VILI [8], including V_T and pressures, and also the respiratory rate (RR). Several equations have been proposed for calculating MP (see Appendix I) [8-12].

1.3 Fully–automated or closed–loop ventilation

INTELLiVENT–ASV is a fully–automated closed–loop mode that automatically controls minute ventilation and oxygenation to reach targets set by the user. It provides either pressure–controlled (i.e., assist) or pressure–support (i.e., assisted) ventilation, depending on patient’s activity. Minute ventilation is adjusted to reach a chosen target for end–tidal CO_2 (etCO_2), in which V_T and RR are adjusted according to respiratory mechanics, including respiratory system compliance (C_{rs}), airway resistance (R_{aw}) and the expiratory time constant (RC_{exp}), and based on the minimal work of breathing principle as described by Otis [13], and the minimal force of breathing as described by Meade [14]. Positive end–expiratory pressure (PEEP) and the oxygen level in inspired air (FiO_2) are adjusted to reach a chosen target for pulse oximetry (SpO_2). In addition, with INTELLiVENT–ASV PEEP is adjusted to reach the lowest ΔP. Thus, with INTELLiVENT–ASV, all components that play a role in VILI are under full control of the ventilator.

1.4 Need for this study

Several studies suggest fully–automated ventilation to ventilate with a lower amount of MP in unselected ICU patients [15], patients after cardiac surgery [16], and patients with and without ARDS [17]. The current study will directly compare the amount of MP in invasively ventilated critically ill patients by calculating MP breath–by–breath, using the various equations proposed in the literature.

2. OBJECTIVE AND HYPOTHESIS

2.1 Objective

To compare INTELLiVENT–adaptive support ventilation (ASV) with conventional ventilation with respect to the ‘Mechanical Power of ventilation’ (MP).

2.2 Hypothesis

INTELLiVENT–ASV compared to conventional ventilation results in a lower MP.

3. STUDY DESIGN

National, multicenter, crossover, randomized clinical trial in invasively ventilated critically ill patients.

4. STUDY POPULATION

4.1 Population

This crossover randomized clinical trial, named ‘INTELLiPOWER’ will recruit consecutive intubated and invasively ventilated critically ill ICU patients who are expected to be ventilated beyond the following calendar day. Patients are included in the ICUs of academic and non–academic centers in the Netherlands. Patients are screened for eligibility and randomized after initial stabilization at INTELLiVENT–ASV, one of the standard modes of ventilation in the participating ICUs.

4.2 Inclusion criteria

- Admitted to one of the participating ICUs;
- Receiving invasive ventilation through a standard endotracheal (i.e., oral) tube;
- Expected to be ventilated > 24 hours; and
- Ventilation is applied by a ventilator that can provide INTELLiVENT–ASV and conventional ventilation.

4.3 Exclusion criteria

- Age under 18 years;
- No written informed consent;
- Morbidly obese; and
- Any contra–indication for use of INTELLiVENT–ASV

4.4 Sample size calculation

Considering a standard deviation of 10.5 J/min (conventional), 90% power, 5% of alpha, a reduction of 3.5 J/min (15% of 24.2 J/min, conventional) and a crossover design, 48 patients in each arm (sequence) is needed (96 in total), ~ 32 at each center. This calculation is based on the results of a non–randomized parallel group study that showed a reduction in MP from 24.2 (± 9.2) to 18.0 (± 11.4) J/min [17]. The report of this study is under review; data are presented in Appendix II.

5. METHODS

5.1 Primary endpoint

The primary endpoint is the amount of MP delivered with each form of invasive ventilation, calculated every 30 minutes using the needed variables for all proposed equations (see Appendix I). Between each ventilation mode, there will be a 30–minutes wash out time.

5.2 Secondary endpoints

Secondary endpoints include:

- Several ventilation parameters (including tidal volume, pressures and respiratory rate)
- Duration of ventilation
- Hospital length of stay (LOS)
- 7- and 28 day-mortality

5.3 Randomization, blinding and treatment allocation

Randomization will be performed using a password protected, SSL–encrypted program (Castor EDC). Randomization sequence is generated by a dedicated computer randomization software program, using random block sizes and is stratified per center. Due to the nature of the treatment, blinding is not possible. Patients are randomly assigned to start with a 3–hour block of conventional ventilation or INTELLiVENT–ASV.

5.4 Study procedures

Patients in participating ICUs are screened and randomized after initial stabilization with conventional ventilation or INTELLiVENT-ASV, choice of ventilatory mode depends on daily care. As soon as possible, but always within 48 hours after start of ventilation in the ICU, patients are then placed in random order for 3 hours at INTELLiVENT–ASV, or at pressure–controlled (called: PCV) or pressure support ventilation (called: SPONT) depending on patient’s activity.

With INTELLiVENT–ASV, the patient’s conditions, ‘ARDS’, ‘Chronic Hypercapnia’ or ‘Traumatic Brain Injury’ can be chosen, if applicable. The controllers for minute volume, PEEP and FiO_2 are all activated, and the target shifts for $etCO_2$ and SpO_2 are adjusted, if necessary.

With conventional ventilation, the same $etCO_2$ and SpO_2 levels are targeted as with INTELLiVENT–ASV, but here the caregiver is fully responsible for choosing the settings.

5.5 Data collection

The data will be collected in Castor Electronic Data Capture (EDC), which meets the criteria of Good Clinical Practice (GCP).

5.5.1 Demographic and general data

- Age (years);
- Gender (male/female);
- Height (cm);
- Weight (kg);
- Comorbidities;
- Date of hospital admission;
- Date of ICU admission;
- Time of ICU admission;
- Time of intubation;
- Reason for ICU admission;
- Cause of respiratory failure;
- Date and time of intubation (if possible, also in transferred patients); and
- Acute Physiology and Chronic Health (APACHE) IV score;

5.5.2 Baseline ventilator settings, variables and parameters to be collected before randomization

- Ventilation mode;
- Tidal volume, inspired (V_{Ti}) (mL);
- Tidal volume, expired (V_{Te}) (mL);
- respiratory rate measured ($RR_{measured}$);
- Maximum airway pressure (Pmax) (cmH_2O);
- Peak pressure (Ppeak) (cmH_2O);
- Plateau pressure (Pplateau) (cmH_2O) (performed with an inspiratory hold);
- Positive end–expiratory pressure (PEEP) (cmH_2O) (performed with an expiratory hold);
- Inspiratory pressure or level of pressure support above PEEP (cmH_2O);
- Tinsp (sec) or set and measured inspiration to expiration ratio (I:E) (ratio);
- Minute volume (MV) (L/min);
- Inspiratory flow (L/min);
- Tslope (sec);

- FiO_2 (%)
- P0.1 (cmH_2O)
- Compliance of the respiratory system (C_{rs}) (L/cmH_2O);
- Resistance of the respiratory system (R_{aw}) (cmH_2O/L/sec);
- End–tidal carbon dioxide (E_tCO_2 (kPa);
- Saturation of peripheral oxygen (SpO2) (%)

5.5.3 Study parameters, also to be collected through an automated data capturing tool

- Ventilation mode;
- Tidal volume set ($V_{T\text{-}set}$) (mL) (only with conventional ventilation);
- Tidal volume, inspired (V_{Ti}) (mL);
- Tidal volume, expired (V_{Te}) (mL);
- respiratory rate set (RR_{set}) (only with conventional ventilation);
- respiratory rate measured ($RR_{measured}$);
- Maximum airway pressure (Pmax) (cmH_2O);
- Peak pressure (Ppeak) (cmH_2O);
- Plateau pressure (Pplateau) (cmH_2O) (performed every 30 minutes with an inspiratory hold);
- Positive end–expiratory pressure (PEEP) (cmH_2O) (performed every 30 minutes with an expiratory hold);
- Inspiratory pressure or level of pressure support above PEEP (cmH_2O);
- Tinsp (sec) or set and measured inspiration to expiration ratio (I:E) (ratio);
- Minute volume (MV) (L/min);
- Inspiratory flow (L/min);
- Tslope (sec);
- FiO_2 (%)
- P0.1 (cmH_2O)
- Compliance of the respiratory system (C_{rs}) (L/cmH_2O);
- Resistance of the respiratory system (R_{aw}) (cmH_2O/L/sec);
- End–tidal carbon dioxide (E_tCO_2 (kPa); and
- Saturation of peripheral oxygen (SpO2) (%)

5.5.4 Blood gas analyses results, if blood gas analyses are performed as part of daily care, within each randomization block

- pH;
- pCO_2 (kPa);
- Bicarbonate (mmol/l);
- pO_2 (kPa); and
- SaO_2 (%)

5.5.5 Follow–up data at day 7 and day 28

- Life status, and if not alive, date of death;
- Location (ICU, hospital, home); and
- Intubation status, if no longer intubated extubation date

5.6 Automatic data collection

The breath–by–breath ventilation data will also be collected using so–called ‘memory boxes’, connected to a free communication port at the ventilator.

5.7 Withdrawal of individual subjects

Subjects can leave the study at any time for any reason if they wish to do so without any consequences. The investigator can decide to withdraw a subject from the study for urgent medical reasons.

5.8Replacement of individual subjects after withdrawal

The calculated number of subjects is intended to be included, i.e., patients who withdraw from the study will be replaced.

5.9 Follow-up of subjects withdrawn from treatment

There will be no follow-up beyond day 28.

5.10 Premature termination of the study

Not applicable.

6. SAFETY REPORTING

6.1 Temporary halt for reasons of subject safety

In accordance to section 10, subsection 4, of the WMO, the sponsor will suspend the study if there is sufficient ground that continuation of the study will jeopardize subject health or safety. The sponsor will notify the accredited METC without undue delay of a temporary halt including the reason for such an action. The study will be suspended pending a further positive decision by the accredited METC. The investigator will take care that all subjects are kept informed.

6.2 Serious adverse events (SAE)

This study compares two ventilation modes with which the teams have extensive experience—both ventilatory modes are seen and used as standard care in the participating intensive care units. Therefore, related serious adverse events (SAEs) or adverse events (AEs) are not expected.

6.3 Follow-up of adverse events

Not applicable.

6.4 Data Safety Monitoring Board (DSMB)

Not applicable.

7. STATISTICAL ANALYSIS

The statistical analysis will be based on an intention–to–treat principle and a per protocol analysis.

Descriptive statistics are first used to study patient characteristics, ventilation parameters and outcomes. Proportions are compared using the chi–squared test or Fisher exact test as required by variable distribution and continuous variables are compared using the Wilcoxon Rank–Sum Test or the Wilcoxon signed–rank test as appropriate. Effects are shown as the average odds ratio (OR) with its 95% confidence interval (95% CI).

To compare the two ventilation strategies, distribution plots showing cumulative rates for V_T, PEEP, Pplat, RR, and mechanical power are constructed. All ventilatory parameters, including V_T, PEEP, Pplat and RR, are presented as medians with their interquartile ranges.

A P value < 0.05 was considered significant.

8. ETHICAL CONSIDERATIONS

8.1 Regulation statement

This study will be conducted according to the principles of the Declaration of Helsinki (revision Fortaleza, Brazil, October 2013) and in accordance with the Medical Research Involving Human Subjects Act (WMO).

8.2 Recruitment and consent

Informed consent will be obtained before any study–related action is taken. Critically ill patients admitted for invasive ventilation to an intensive care unit are, without exception, incompetent to give informed consent, and so a legal representative will be asked for the informed consent. Re–consent is asked when the patient is adequately awake.

8.3 Benefits and risks assessment, group relatedness

Not applicable.

8.4 Compensation for injury

The sponsor/investigator and the studysites have a liability insurance which is in accordance with article 7 of the WMO. As this study compares two ventilation strategies used for standard care, an exception form the requirement for insurance to cover for damage to research objects through injury or death caused by the study is applicable.

9. ADMINISTRATIVE ASPECTS, MONITORING AND PUBLICATION

9.1 Handling and storage of data and documents

Subject data will be stored in a pseudonymized form, which means only local investigators will have access to the key to the code that relates the individual data to identifiable patients. Used data as written in the case report form will not contain any identifiable or relatable data. All handling of personal data will comply with the General Data Protection Regulation and the 'Reuse of care data for the purpose of research' standard of the AMC.

9.2 Monitoring and Quality Assurance

Queries on the database will be done and analyzed by the monitor to signalize early aberrant patterns, trends, issues with consistency of credibility and other anomalies. On site monitoring will compromise controlling presence and completeness of the research dossier and the informed consent forms, source data checks will be performed as described in the monitoring plan. A monitoring plan is being developed.

9.3 Amendments

Not applicable.

9.4 Annual progress report

Not applicable.

9.5 Temporary halt and (prematurely) end of study report

Not applicable.

9.6 Public disclosure and publication policy

The study protocol will be registered before inclusion of the first patient at clinicaltrials.gov. The results of the study will find their way into (inter–)national scientific journals and guidelines. We will submit analyses to scientific journals in the field of intensive care medicine.

10. REFERENCES

1. Acute Respiratory Distress Syndrome, N., et al., Ventilation with lower tidal volumes as compared with traditional tidal volumes for acute lung injury and the acute respiratory distress syndrome. N Engl J Med, 2000. 342(18): p. 1301-8.
2. Fan, E., et al., An Official American Thoracic Society/European Society of Intensive Care Medicine/Society of Critical Care Medicine Clinical Practice Guideline: Mechanical Ventilation in Adult Patients with Acute Respiratory Distress Syndrome. Am J Respir Crit Care Med, 2017. 195(9): p. 1253-1263.
3. Amato, M.B., et al., Driving pressure and survival in the acute respiratory distress syndrome. N Engl J Med, 2015. 372(8): p. 747-55.
4. Esteban, A., et al., Evolution of mortality over time in patients receiving mechanical ventilation. Am J Respir Crit Care Med, 2013. 188(2): p. 220-30.
5. Protti A, Andreis DT, Monti M, Santini A, Sparacino CC, Langer T, Votta E, Gatti S, Lombardi L, Leopardi O, Masson S, Cressoni M, Gattinoni L (2013) Lung stress and strain during mechanical ventilation: any difference between statics and dynamics? Crit Care Med. 2013 Apr;41(4):1046-55
6. Cressoni M, Gotti M, Chiurazzi C, Massari D, Algieri I, Amini M, Cammaroto A, Brioni M, Montaruli C, Nikolla K, Guanziroli M, Dondossola D, Gatti S, Valerio V, Vergani GL, Pugni P, Cadringher P, Gagliano N, Gattinoni L (2016). Mechanical power and development of ventilator-induced lung injury. Anesthesiology 124:1100–1108
7. Serpa Neto, A., Deliberato, R. O., Johnson, A. E. W., Bos, L. D., Amorim, P., Pereira, S. M., Cazati, D. C., Cordioli, R. L., Correa, T. D., Pollard, T. J., Schettino, G. P. P., Timenetsky, K. T., Celi, L. A., Pelosi, P., Gama de Abreu, M., & Schultz, M. J. (2018). Mechanical power of ventilation is associated with mortality in critically ill patients: an analysis of patients in two observational cohorts. Intensive Care Medicine, 44(11), 1914–1922.
8. Giosa, L., Busana, M., Pasticci, I., Bonifazi, M., Macrì, M. M., Romitti, F., Vassalli, F., Chiumello, D., Quintel, M., Marini, J. J., & Gattinoni, L. (2019). Mechanical power at a glance: a simple surrogate for volume-controlled ventilation. Intensive Care Medicine Experimental, 7(1).
9. Gattinoni, L., Tonetti, T., Cressoni, M., Cadringher, P., Herrmann, P., Moerer, O., Protti, A., Gotti, M., Chiurazzi, C., Carlesso, E., Chiumello, D., & Quintel, M.

(2016). Ventilator-related causes of lung injury: the mechanical power. Intensive Care Medicine, 42(10), 1567–1575.

10. van der Meijden, S., Molenaar, M., Somhorst, P., & Schoe, A. (2019). Calculating mechanical power for pressure-controlled ventilation. Intensive Care Medicine, 45(10), 1495–1497.
11. Becher, T., van der Staay, M., Schädler, D., Frerichs, I., & Weiler, N. (2019). Calculation of mechanical power for pressure-controlled ventilation. Intensive Care Medicine, 45(9), 1321–1323.
12. Silva, P. L., Ball, L., Rocco, P. R. M., & Pelosi, P. (2019). Power to mechanical power to minimize ventilator-induced lung injury? Intensive Care Medicine Experimental, 7(S1), 1–11.
13. Otis, A.B., Fenn, W.O., Rahn, H. (1950). Mechanics of breathing in man. J Appl Physiol; 2: 592-607
14. Mead J.: Control of respiratory frequency. J Appli Physiol 1960; 15: pp. 325-336
15. Arnal, J.M., Saoli, M., Garnero, A. Airway and transpulmonary driving pressures and mechanical powers selected by INTELLiVENT-ASV in passive, mechanically ventilated ICU patients (2019) Heart Lung. S0147-9563(19)30533-3.
16. The POSITiVE-investigators, de Bie, A.J., Serpa Neto, A., van Meenen, D.M., Bouwman. A.R., Roos, A.N., Korsten, E.H., Lameijer, J.R., Bindels, A.G., Schultz, M.J. (2020). Effect of a closed–loop versus conventional ventilation strategy on proportion of time in unacceptable ventilation zones in patients after cardiac surgery – a randomized clinical trial. BJA, 125(5): 739-749
17. Buiteman-Kruizinga, L.A., Mkadmi, H.E., Schultz, M.J., Tangkau, P.L., van der Heiden, P.L.J. (2021). The effect of closed–loop ventilation on mechanical power – results from a pilot study of adaptive support ventilation. *In press; Critical Care Explorations*

APPENDIX I

Equations for MP to be used:

$$MP = 0.098 * RR * \{\Delta V^2 \times [½ * EL_{rs} + RR * \frac{(1+I:E)}{60 \times I:E} * R_{aw}] + \Delta V * PEEP\}$$ [Eq. 1] [9]

$$MP = 0.098 * V_T * RR * (Ppeak – ½ * \Delta P)$$ [Eq. 2] [9]

$$MP = \frac{\text{minute volume} * (\text{peak pressure (Ppeak)} + \text{PEEP} + \frac{F}{6})}{20}$$ [Eq. 3] [8]

$$MP = 0.098 * RR * V_t \cdot \left(PEEP + P_{insp} \cdot \left(1 - e^{\frac{-T_{insp}}{\tau}}\right)\right)$$ [Eq. 4] [11]

$$MP = 0.098 * RR(PEEP + \Delta P_{insp}) * V_t - \Delta P_{insp}{}^2\, C * \left(0.5 - \frac{\tau}{T_{slope}} + \frac{\tau^2}{T_{slope^2}}\left(1 - e^{\frac{T_{slope}}{\tau}}\right)\right)$$ [Eq. 5] [10]

$$MP = 0.098 * RR * V_T * (\Delta Pinsp + PEEP)$$ [Eq. 6] [11]

$$MP = V_T * \Delta P_{RS} * RR$$ [Eq. 7] [12]

APPENDIX II

Pilot study data:

N=24

Conventional ventilation N=12

	Mean	Median	Standard Deviation	Percentile 25	Percentile 75
MP	24.28	22.94	9.23	16.36	30.63

Closed-loop ventilation N=12

	Mean	Median	Standard Deviation	Percentile 25	Percentile 75
MP	18.00	15.08	11.42	11.73	19.82
