# Supplementary material for: Effect of automated versus conventional ventilation on mechanical power of ventilation—A randomized crossover clinical trial
Source: PLoS One. 2024 Jul 30;19(7):e0307155. doi: 10.1371/journal.pone.0307155 (PMC11288413; doi:10.1371/journal.pone.0307155)
Supplement: S2 Fig — (DOCX) [file pone.0307155.s004.docx]

*
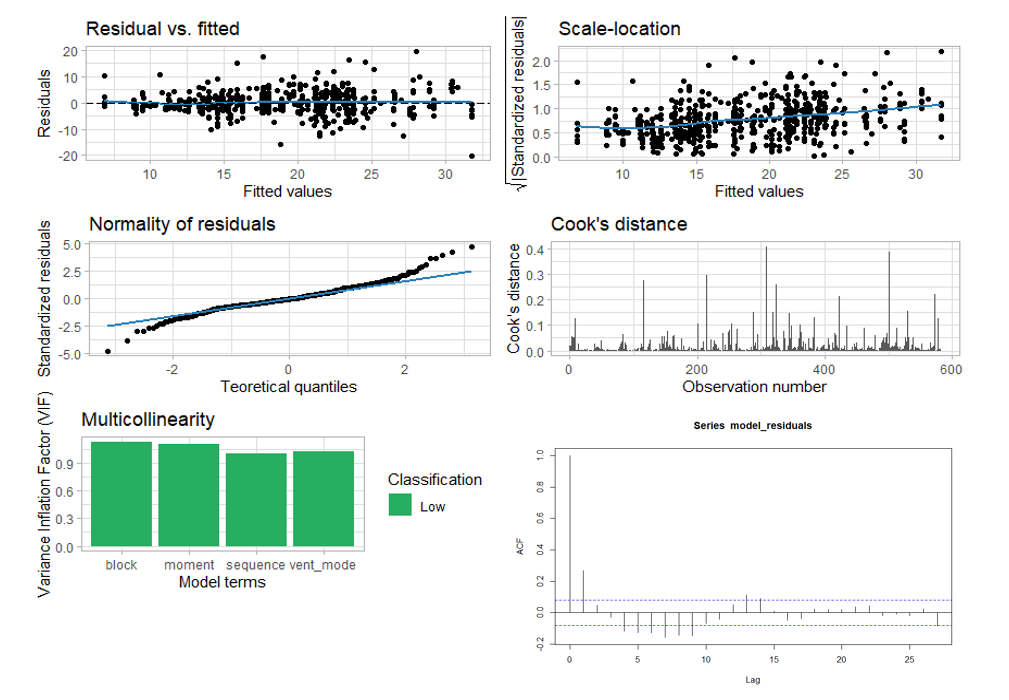
*

***eFigure S2****. Figures of the residual analysis, of the mixed-effect generalized linear model including only passive patients*
