# Supplementary material for: Effect of automated versus conventional ventilation on mechanical power of ventilation—A randomized crossover clinical trial
Source: PLoS One. 2024 Jul 30;19(7):e0307155. doi: 10.1371/journal.pone.0307155 (PMC11288413; doi:10.1371/journal.pone.0307155)
Supplement: S4 Fig — Distribution plots of ventilatory parameters in passive patients, showing all measurements of every patient. Vertical dotted lines represent the median value with conventional ventilation. Horizontal dotted lines show the respective proportion of patients reaching each cutoff. (DOCX) [file pone.0307155.s006.docx]

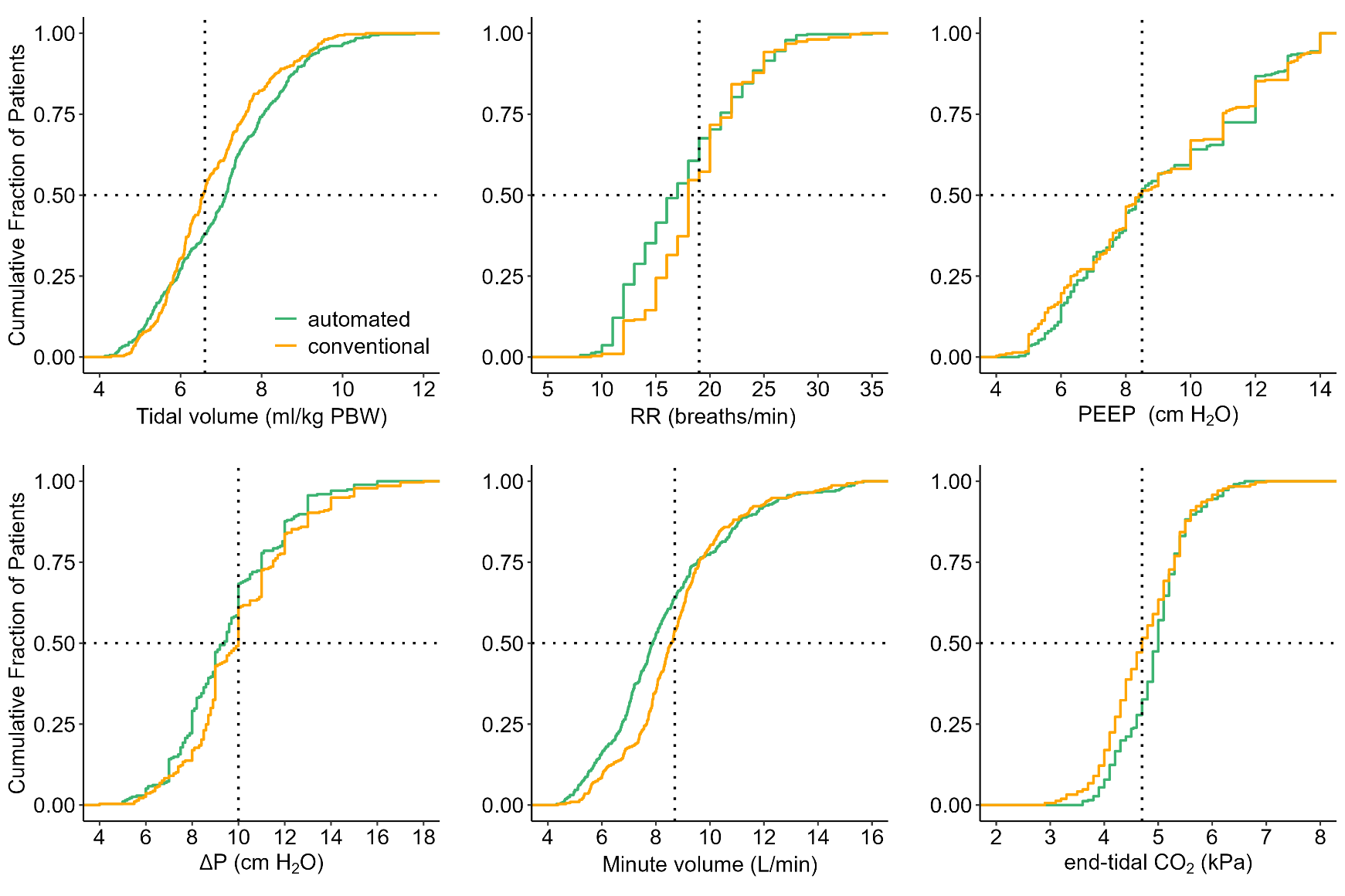


**Figure S4**. Distribution plots of ventilatory parameters in passive patients, showing all measurements of every patient. Vertical dotted lines represent the median value with conventional ventilation. Horizontal dotted lines show the respective proportion of patients reaching each cutoff.
