# Supplementary material for: Effect of automated versus conventional ventilation on mechanical power of ventilation—A randomized crossover clinical trial
Source: PLoS One. 2024 Jul 30;19(7):e0307155. doi: 10.1371/journal.pone.0307155 (PMC11288413; doi:10.1371/journal.pone.0307155)
Supplement: S1 Table — Ventilatory parameters, conventional ventilation before randomization (n = 34). (DOCX) [file pone.0307155.s008.docx]

| **Table S1. Ventilatory parameters, conventional ventilation before randomization (n = 34)** | | | | |
| --- | --- | --- | --- | --- |
|  | automated  ventilation | conventional ventilation | mean difference  (95% CI) | *p* |
| *Primary endpoint* |  |  |  |  |
| MP, median [IQR] and mean (SD) (J/min) | 14.3 [11.7–21.2]  16.4 (6.2) | 19.1 [14.8–24.6]  19.5 (6.7) | –3.33 (–4.42 to –2.22) | < 0.01 |
| *Ventilation variables and parameters* | | | | |
| V_Ti_ (mL) | 500 [414–575] | 461 [410–533] | 25.38 (7.84 to 42.94) | 0.01 |
| V_Te_ (mL) | 499 [429–570] | 466 [406–536] | 18.00 (–0.073 to 36.78) | 0.04 |
| V_T_ (ml/kg PBW) | 7.3 [6.2–8.4] | 6.7 [5.8–8.2] | 0.36 (0.09 to 0.64) | 0.01 |
| RR (breaths/minute) | 16 [13–20] | 18 [16–22] | –2.52 (–3.29 to –1.74) | < 0.01 |
| Minute volume (cm H_2_O) | 7.8 [6.4–10.3] | 8.7 [7.7–10.3] | –0.80 (–1.25 to –0.35) | < 0.01 |
| Pmax (cm H_2_O) | 21 [18–25] | 21 [17–26] | –0.02 (–0.76 to 0.73) | ns |
| PEEP, set (cm H_2_O) | 8 [6–10] | 8 [6–10] | 0.22 (–0.11 to 0.54) | ns |
| Pinsp (cm H_2_O) + | 12 [10–14] | 14 [12–15] | –1.08 (–1.57 to –0.59) | < 0.01 |
| PS (cm H_2_O) * | 1 [7–16] | 10 [6–14] | 0.94 (0.16 to 1.73) | 0.02 |
| ΔP, dynamic (cm H_2_O) | 13 [10–15] | 13 [11–16] | –0.18 (–0.70 to 0.34) | ns |
| Flow (L/min) | 40.7 [34.3–49.7] | 41.6 [34.5–50.3] | –0.25 (–1.49 to 0.99) | ns |
| Tinsp _(_sec) | 1.15 [0.97–1.47] | 1.08 [0.95–1.2] | 0.16 (0.12 to 0.21) | < 0.01 |
| FiO_2_ (%) | 32 [28–38] | 35 [26–40] | –1.13 (–2.65 to 0.38) | ns |
| etCO_2_ (kPa) | 4.9 [4.4–5.3] | 4.9 [4.2–5.6] | 0.06 (–0.02 to 0.14) | ns |
| SpO_2_ (%) | 94 [93–96] | 95 [94–96] | –0.04 (–0.75 to –0.09) | 0.01 |
| C_RS_ (mL/cm H_2_O) | 40.6 [31.3–52.6] | 36.9 [29.7–46.6] | 0.28 (–3.72 to 4.28) | ns |
| Values are median [IQR] or mean (SD).  Abbreviations:mL, milliliter; cm H_2_O, centimeters of water; L, liter; sec, seconds; kPa, kilopascal; J/min, joule per minute; MP, mechanical power; V_T_, tidal volume; RR, respiratory rate; Pmax, maximum airway pressure; PEEP, positive end–expiratory pressure; Pinsp, set inspiratory pressure; PS, set pressure support; ΔP, driving pressure; Tinsp, inspiratory time; FiO_2_, fraction of inspired oxgen; etCO_2_, end–tidal carbon dioxide; SpO_2_, pulse oximetry; C_RS_, compliance of the respiratory system; CI, confidence interval.  ^+^ available in passive patients; * available in active patients | | | | |
