# Supplementary material for: Effect of automated versus conventional ventilation on mechanical power of ventilation—A randomized crossover clinical trial
Source: PLoS One. 2024 Jul 30;19(7):e0307155. doi: 10.1371/journal.pone.0307155 (PMC11288413; doi:10.1371/journal.pone.0307155)
Supplement: S2 Table — Ventilatory parameters in passive patients, conventional ventilation before randomization. (DOCX) [file pone.0307155.s009.docx]

| **Table S2. Ventilatory parameters in passive patients, conventional ventilation before randomization (n = 29)** | | | | | |
| --- | --- | --- | --- | --- | --- |
|  | automated  ventilation | conventional ventilation (PCV) | mean difference  (95% CI) | *p* | |
| *Primary endpoint* |  |  |  | |  |
| MP, median [IQR] and mean (SD) (J/min) | 14.3 [11.7–21.2]  16.4 (6.2) | 19.2 [14.7–24.8]  19.5 (6.7) | –3.10 (–4.19 to –2.00) | | < 0.01 |
| *Ventilation variables and parameters* | | | | | |
| V_Ti_ (mL) | 500 [408–572] | 446 [396–492] | 42.08 (21.66 to 62.77) | | < 0.01 |
| V_Te_ (mL) | 489 [416–557] | 455 [396–501] | 29.03 (7.71 to 50.82) | | 0.01 |
| V_T_ (ml/kg PBW) | 7.3 [6.0–7.9] | 6.2 [5.6–7.6] | 0.57 (0.26 to 0.88) | | 0.01 |
| RR (breaths/minute) | 16 [14–18] | 18 [17–22] | –3.97 (–4.60 to –3.34) | | < 0.01 |
| Minute volume (cm H_2_O) | 7.5 [6.0–9.3] | 8.5 [7.8–9.4] | –1.12 (–1.53 to –0.71) | | < 0.01 |
| Pmax (cm H_2_O) | 21 [18–25] | 23 [18–28] | –0.81 (–1.75 to 0.14) | | ns |
| PEEP, set (cm H_2_O) | 9 [6–10] | 9 [6–10] | 0.28 (–0.15 to 0.72) | | ns |
| Pinsp (cm H_2_O) | 12 [10–14] | 14 [12–15] | –1.16 (–1.61 to –0.61) | | < 0.01 |
| ΔP, static (cm H_2_O) | 10 [8–12] | 10 [9–12] | –0.13 (–0.67 to 0.43) | | ns |
| FiO_2_ (%) | 33 [29–38] | 35 [30–40] | –2.37 (–4.58 to –0.13) | | 0.04 |
| etCO_2_ (kPa) | 4.9 [4.4–5.2] | 5.0 [4.0–5.7] | 0.16 (0.06 to 0.26) | | 0.01 |
| SpO_2_ (%) | 94 [93–96] | 95 [94–96] | –0.84 (–1.32 to –0.35) | | < 0.01 |
| C_RS_ (mL/cm H_2_O) | 38.5 [31.2–47.4] | 33.4 [27.0–38.0] | 6.35 (3.63 to 9.11) | | < 0.01 |
| Values are median [IQR] or mean (SD).  Abbreviations:mL, milliliter; cm H_2_O, centimeters of water; L, liter; sec, seconds; kPa, kilopascal; J/min, joule per minute; MP, mechanical power; V_T_, tidal volume; RR, respiratory rate; Pmax, maximum airway pressure; PEEP, positive end–expiratory pressure; Pinsp, set inspiratory pressure; PS, set pressure support; ΔP, driving pressure; FiO_2_, fraction of inspired oxgen; etCO_2_, end–tidal carbon dioxide; SpO_2_, pulse oximetry; C_RS_, compliance of the respiratory system; PCV, pressure–controlled ventilation | | | | | |
