# Supplementary material for: Effect of automated versus conventional ventilation on mechanical power of ventilation—A randomized crossover clinical trial
Source: PLoS One. 2024 Jul 30;19(7):e0307155. doi: 10.1371/journal.pone.0307155 (PMC11288413; doi:10.1371/journal.pone.0307155)
Supplement: S3 Table — Ventilatory parameters, automated ventilation before randomization (n = 62). (DOCX) [file pone.0307155.s010.docx]

| **Table S3. Ventilatory parameters, automated ventilation before randomization (n = 62)** | | | | |
| --- | --- | --- | --- | --- |
|  | automated  ventilation | conventional ventilation | mean difference  (95% CI) | *p* |
| *Primary endpoint* |  |  |  |  |
| MP, median [IQR] and mean (SD) (J/min) | 14.4 [10.8–19.6]  16.1 (7.8) | 15.9 [11.3–21.3]  17.0 (8.0) | –0.43 (–1.41 to 0.55) | ns |
| *Ventilation variables and parameters* | | | | |
| V_Ti_ (mL) | 474 [396–590] | 487 [415–549] | –3.78 (–16.56 to 9.02) | ns |
| V_Te_ (mL) | 478 [402–586] | 497 [430–563] | –5.11 (–17.55 to 7.34) | ns |
| V_T_ (ml/kg PBW) | 7.0 [5.8–8.1] | 7.0[ 6.1–7.9] | –0.09 (–0.28 to 0.10) | ns |
| RR (breaths/minute) | 18 [14–23] | 18 [15–23] | –0.42 (–1.01 to 0.17) | ns |
| Minute volume (cm H_2_O) | 8.5 [7.0–10.6] | 8.8 [7.4–10.5] | –0.35 (–0.69 to –0.02) | 0.04 |
| Pmax (cm H_2_O) | 22 [18–25] | 21 [17–24] | 0.26 (–0.28 to 0.79) | ns |
| PEEP, set (cm H_2_O) | 8 [6–12] | 8 [6–10] | 0.13 (–0.12 to 0.38) | ns |
| Pinsp (cm H_2_O) | 12 [10–16] | 13 [11–15] | 0.18 (–0.26 to 0.62) | ns |
| ΔP, dynamic (cm H_2_O) | 12 [10–15] | 13 [10–16] | 0.13 (–0.35 to 0.60) | ns |
| Flow (L/min) | 44.7 [37.5–53] | 46.9 [39.9–55.9] | –2.80 (–3.92 to –1.68) | < 0.01 |
| Tinsp _(_sec) | 1.07 [0.81–1.38] | 1.00 [0.8–1.25] | 0.09 (–0.07 to 0.13) | < 0.01 |
| FiO_2_ (%) | 31 [29–38] | 30 [28–10] | –0.70 (–1.54 to 0.14) | ns |
| etCO_2_ (kPa) | 4.9 [4.4–5.3] | 4.7 [4.3–5.2] | 0.12 (0.07 to 0.17) | < 0.01 |
| SpO_2_ (%) | 94 [92–96] | 95 [92–97] | –0.31 (–0.65 to 0.01) | ns |
| C_RS_ (mL/cm H_2_O) | 36.3 [29.8–48.5] | 37.0 [29.3–53.3] | –2.15 (–4.75 to 0.45) | ns |
| Values are median [IQR] or mean (SD).  Abbreviations:mL, milliliter; cm H_2_O, centimeters of water; L, liter; sec, seconds; kPa, kilopascal; J/min, joule per minute; MP, mechanical power; V_T_, tidal volume; RR, respiratory rate; Pmax, maximum airway pressure; PEEP, positive end–expiratory pressure; Pinsp, set inspiratory pressure; PS, set pressure support; ΔP, driving pressure; Tinsp, inspiratory time; FiO_2_, fraction of inspired oxgen; etCO_2_, end–tidal carbon dioxide; SpO_2_, pulse oximetry; C_RS_, compliance of the respiratory system; CI, confidence interval. | | | | |
