# Supplementary material for: Effect of automated versus conventional ventilation on mechanical power of ventilation—A randomized crossover clinical trial
Source: PLoS One. 2024 Jul 30;19(7):e0307155. doi: 10.1371/journal.pone.0307155 (PMC11288413; doi:10.1371/journal.pone.0307155)
Supplement: S4 Table — Ventilatory parameters in passive patients, automated ventilation before randomization (n = 56). (DOCX) [file pone.0307155.s011.docx]

| **Table S4. Ventilatory parameters in passive patients, automated ventilation before randomization (n=56)** | | | | |
| --- | --- | --- | --- | --- |
|  | automated  ventilation | conventional ventilation (PCV) | mean difference  (95% CI) | *p* |
| *Primary endpoint* |  |  |  |  |
| MP, median [IQR] and mean (SD) (J/min) | 18.2 [13.7–24.6]  19.8 (9.5) | 19.1 [13.6–24.0]  19.2 (7.0) | –0.31 (–1.38 to 0.76) | ns |
| *Ventilation variables and parameters* | | | | |
| V_Ti_ (mL) | 469 [393–586] | 485 [426–529] | 9.89 (–2.67 to 22.38) | ns |
| V_T_ (ml/kg PBW) | 7.0 [5.8–8.0] | 6.8 [6.1–7.6] | 0.11 (–0.07 to 0.28) | ns |
| RR (breaths/minute) | 18 [14–22] | 18 [15–20] | –0.42 (–1.09 to 0.25) | ns |
| Minute volume (cm H_2_O) | 8.2 [6.9–10.5] | 8.5 [7.4–9.7] | –0.16 (–0.53 to 0.21) | ns |
| Pplat (cm H_2_O) | 22 [19–26] | 22 [19–25] | 0.07 (–0.68 to 0.82) | ns |
| PEEP, set (cm H_2_O) | 8 [6–12] | 7 [6–10] | 0.10 (–0.20 to 0.40) | ns |
| Pinsp (cm H_2_O) | 12 [10–16] | 13 [11–15] | –0.01 (–0.54 to 0.28) | ns |
| ΔP, static (cm H_2_O) | 9 [8–11] | 10 [9–12] | –0.49 (–0.83 to –0.15) | 0.01 |
| FiO_2_ (%) | 31 [30–38] | 30 [28–40] | –0.73 (–2.03 to 0.57) | ns |
| etCO_2_ (kPa) | 5.0 [4.5–5.4] | 4.7 [4.3–5.2] | 0.12 (0.05 to 0.20) | 0.01 |
| SpO_2_ (%) | 95 [92–97] | 96 [93–97] | –0.22 (–0.58 to 0.14) | ns |
| C_RS_ (mL/cm H_2_O) | 35.1 [29.0–44.0] | 33.0 [27.3–42.4] | 2.5 (0.17 to 4.82) | 0.04 |
| Values are median [IQR] or mean (SD).  Abbreviations:mL, milliliter; cm H_2_O, centimeters of water; L, liter; sec, seconds; kPa, kilopascal; J/min, joule per minute; MP, mechanical power; V_T_, tidal volume; RR, respiratory rate; Pmax, maximum airway pressure; PEEP, positive end–expiratory pressure; Pinsp, set inspiratory pressure; PS, set pressure support; ΔP, driving pressure; FiO_2_, fraction of inspired oxgen; etCO_2_, end–tidal carbon dioxide; SpO_2_, pulse oximetry; C_RS_, compliance of the respiratory system; PCV, pressure–controlled ventilation; CI, confidence interval. | | | | |
