# Supplementary material for: Effect of automated versus conventional ventilation on mechanical power of ventilation—A randomized crossover clinical trial
Source: PLoS One. 2024 Jul 30;19(7):e0307155. doi: 10.1371/journal.pone.0307155 (PMC11288413; doi:10.1371/journal.pone.0307155)
Supplement: S5 Table — Ventilatory parameters in patients with CRS ≤ 37.3 (n = 50). (DOCX) [file pone.0307155.s012.docx]

| **Table S5. Ventilatory parameters in patients with C_RS_ ≤ 37.3 (n = 50)** | | | | |
| --- | --- | --- | --- | --- |
|  | automated  ventilation | conventional ventilation | mean difference  (95% CI) | *p* |
| *Primary endpoint* |  |  |  |  |
| MP, median [IQR] and mean (SD) (J/min) | 18.9 [13.7–24.6]  19.5 (9.0) | 20.5 [15.8–25.6]  20.6 (6.8) | –1.13 (–2.16 to –0.10) | 0.03 |
| *Ventilation variables and parameters* | | | | |
| V_Ti_ (mL) | 452 [377–519] | 455 [392–512] | –0.09 (–13.08 to 12.91) | ns |
| V_Te_ (mL) | 462 [369–534] | 469 [414–529] | 0.52 (–12.11 to 13.18) | ns |
| V_T_ (ml/kg PBW) | 6.8 [5.6–7.6] | 6.6 [5.8–7.7] | –0.03 (–0.22 to 0.16) | ns |
| RR (breaths/minute) | 19 [15–23] | 20 [17–22] | –0.82 (–1.46 to –0.18) | 0.01 |
| Minute volume (cm H_2_O) | 8.1 [6.7–10.5] | 8.8 [7.6–103] | –0.28 (–1.03 to 0.48) | 0.03 |
| Pmax (cm H_2_O) | 23 [20–27] | 23 [20–27] | –0.41 (–0.99 to 0.18) | ns |
| PEEP, set (cm H_2_O) | 9 [6–12] | 9 [7–12] | –0.04 (–0.31 to 0.23) | ns |
| Pinsp (cm H_2_O) | 13 [11–16] | 14 [12–15] | –0.12(–0.53 to 0.30) | ns |
| ΔP, dynamic (cm H_2_O) | 14 [11–17] | 14 [12–17] | –0.37 (–0.88 to 0.15) | ns |
| Flow (L/min) | 43.5 [35.5–52.1] | 45.7 [38.9–53.1] | –1.89 (–2.98 to –0.80) | < 0.01 |
| FiO_2_ (%) | 33.5 [30–40] | 35 [30–45] | –1.44 (–2.57 to –0.31) | 0.01 |
| etCO_2_ (kPa) | 4.9 [4.5–5.3] | 4.8 [4.3–5.4] | 0.07 (0.01 to 0.13) | 0.02 |
| SpO_2_ (%) | 94 [92–96] | 95 [93–97] | –0.65 (–0.93 to –0.38) | < 0.01 |
| C_RS_ (mL/cm H_2_O) | 32.6 [27.1–39.3] | 31.4 [27.0–37.0] | 1.33 (–0.65 to 3.30) | ns |
| Values are median [IQR] or mean (SD).  Abbreviations:mL, milliliter; cm H_2_O, centimeters of water; L, liter; sec, seconds; kPa, kilopascal; J/min, joule per minute; MP, mechanical power; V_T_, tidal volume; RR, respiratory rate; Pmax, maximum airway pressure; PEEP, positive end–expiratory pressure; Pinsp, set inspiratory pressure; PS, set pressure support; ΔP, driving pressure; Tinsp, inspiratory time; FiO_2_, fraction of inspired oxgen; etCO_2_, end–tidal carbon dioxide; SpO_2_, pulse oximetry; C_RS_, compliance of the respiratory system; CI, confidence interval. | | | | |
