# Supplementary material for: Effect of automated versus conventional ventilation on mechanical power of ventilation—A randomized crossover clinical trial
Source: PLoS One. 2024 Jul 30;19(7):e0307155. doi: 10.1371/journal.pone.0307155 (PMC11288413; doi:10.1371/journal.pone.0307155)
Supplement: S6 Table — Ventilatory parameters in passive patients with CRS ≤ 37.3 (n = 32). (DOCX) [file pone.0307155.s013.docx]

| **Table S6. Ventilatory parameters in passive patients with C_RS_ ≤ 37.3 (n = 32)** | | | | |
| --- | --- | --- | --- | --- |
|  | automated  ventilation | conventional ventilation (PCV) | mean difference  (95% CI) | *p* |
| *Primary endpoint* |  |  |  |  |
| MP, median [IQR] and mean (SD) (J/min) | 18.4 [13.8–24.2]  18.8 (7.2) | 20.7 [16.0–26.2]  21.1 (6.8) | –1.74 (–2.69 to –0.79) | < 0.01 |
| *Ventilation variables and parameters* | | | | |
| V_Ti_ (mL) | 474 [374–525] | 449 [388–503] | 18.54 (4.94 to 32.12) | < 0.01 |
| V_Te_ (mL) | 466 [389–540] | 460 [402–526] | 17.15 (2.80 to 31.44) | 0.02 |
| V_T_ (ml/kg PBW) | 7.0 [5.5–7.8] | 6.6 [5.7–7.6] | 0.26 (0.05 to 0.47) | 0.02 |
| RR (breaths /minute) | 18 [15–23] | 20 [18–22] | –2.05 (–2.63 to –1.46) | < 0.01 |
| Minute volume (cm H_2_O) | 7.8 [6.4–10.2] | 8.7 [7.6–10.0] | –0.63 (–0.95 to –0.32) | < 0.01 |
| Pplat (cm H_2_O) | 23 [20–27] | 24 [21–28] | 0.07 (–0.68 to 0.82) | ns |
| PEEP, set (cm H_2_O) | 9 [7–12] | 9 [7–12] | –0.06 (–0.36 to 0.23) | ns |
| Pinsp (cm H_2_O) | 13 [11–16] | 14 [12–15] | –0.37 (–0.75 to 0.002) | ns |
| ΔP, static (cm H_2_O) | 10 [8–12] | 10 [9–12] | –0.58 (–0.95 to –0.21) | 0.01 |
| FiO_2_ (%) | 34 [30–40] | 35 [30–45] | –1.51 (–3.15 to 0.13) | ns |
| etCO_2_ (kPa) | 4.9 [4.6–5.3] | 4.7 [4.3–5.4] | 0.13 (0.06 to 0.21) | < 0.01 |
| SpO_2_ (%) | 94 [92–96] | 95 [93–97] | –0.77 (–1.13 to –0.41) | < 0.01 |
| C_RS_ (mL/cm H_2_O) | 32.6 [27.3–37.2] | 30.0 [26.0–34.4] | 3.25 (1.32 to 5.17) | < 0.01 |
| Values are median [IQR] or mean (SD).  Abbreviations:mL, milliliter; cm H_2_O, centimeters of water; L, liter; sec, seconds; kPa, kilopascal; J/min, joule per minute; MP, mechanical power; V_T_, tidal volume; RR, respiratory rate; Pmax, maximum airway pressure; PEEP, positive end–expiratory pressure; Pinsp, set inspiratory pressure; PS, set pressure support; ΔP, driving pressure; Tinsp, inspiratory time; FiO_2_, fraction of inspired oxgen; etCO_2_, end–tidal carbon dioxide; SpO_2_, pulse oximetry; C_RS_, compliance of the respiratory system; PCV, pressure–controlled ventilation; CI, confidence interval. | | | | |
