# Supplementary material for: Effect of automated versus conventional ventilation on mechanical power of ventilation—A randomized crossover clinical trial
Source: PLoS One. 2024 Jul 30;19(7):e0307155. doi: 10.1371/journal.pone.0307155 (PMC11288413; doi:10.1371/journal.pone.0307155)
Supplement: S7 Table — Ventilatory parameters in patients with CRS > 37.3 (n = 46). (DOCX) [file pone.0307155.s014.docx]

| **Table S7. Ventilatory parameters in patients with C_RS_ > 37.3 (n = 46)** | | | | |
| --- | --- | --- | --- | --- |
|  | automated ventilation | conventional ventilation | mean difference  (95% CI) | *p* |
| *Primary endpoint* |  |  |  |  |
| MP, median [IQR] and mean (SD) (J/min) | 14.5 [10.6–19.6]  15.8 (6.9) | 14.4 [9.9–22.1]  16.2 (7.7) | –0.10 (–1.04 to 0.83) | ns |
| *Ventilation variables and parameters* | | | | |
| V_Ti_ (mL) | 541 [445–638] | 506 [429–581] | 16.58 (1.84 to 31.33) | 0.03 |
| V_Te_ (mL) | 524 [436–614] | 499 [436–593] | 7.45 (–7.87 to 22.78) | ns |
| V_T_ (ml/kg PBW) | 7.4 [6.6–8.6] | 7.1 [6.3–8.5] | 0.21 (–0.02 to 0.43) | 0.06 |
| RR (breaths/minute) | 16 [13–21] | 17 [15–22] | –1.26 (–1.90 to –0.63) | < 0.01 |
| Minute volume (cm H_2_O) | 8.4 [7.0–10.6] | 8.8 [7.4–10.6] | –0.37 (–0.70 to –0.04) | 0.03 |
| Pmax (cm H_2_O) | 19 [16–22] | 18 [15–22] | 0.70 (0.13 to 1.27) | 0.02 |
| PEEP, set (cm H_2_O) | 8 [6–10] | 7 [5–10] | 0.29 (0.03 to 0.54) | 0.03 |
| Pinsp (cm H_2_O) | 11 [8–14] | 12 [10–15] | –0.26 (–0.75 to 0.23) | ns |
| ΔP, dynamic (cm H_2_O) | 11 [9–13] | 11 [8–14] | 0.41 (–0.07 to 0.89) | ns |
| Flow (L/min) | 44.0 [37.5–53.1] | 42.8 [35.5–53.6] | –0.94 (–2.15 to 0.27) | ns |
| FiO_2_ (%) | 30 [26–35] | 30 [25–39] | 0.20 (–0.68 to 1.10) | ns |
| etCO_2_ (kPa) | 5.0 [4.3–5.4] | 4.8 [4.2–5.3] | 0.11 (0.05 to 0.16) | < 0.01 |
| SpO_2_ (%) | 93 [95–97] | 95 [93–97] | –0.08 (–0.45 to 0.29) | ns |
| C_RS_, dynamic (mL/cm H_2_O) | 47.2 [37.7–63.0] | 43.7 [37.0–61.9] | 4.86 (–8.59 to –1.22) | 0.01 |
| Values are median [IQR] or mean (SD).  Abbreviations: mL, milliliter; cm H_2_O, centimeters of water; L, liter; sec, seconds; kPa, kilopascal; J/min, joule per minute; MP, mechanical power; V_T_, tidal volume; RR, respiratory rate; Pmax, maximum airway pressure; PEEP, positive end–expiratory pressure; Pinsp, set inspiratory pressure; PS, set pressure support; ΔP, driving pressure; Tinsp, inspiratory time; FiO_2_, fraction of inspired oxgen; etCO_2_, end–tidal carbon dioxide; SpO_2_, pulse oximetry; C_RS_, compliance of the respiratory system; CI, confidence interval. | | | | |
