# Supplementary material for: Effect of automated versus conventional ventilation on mechanical power of ventilation—A randomized crossover clinical trial
Source: PLoS One. 2024 Jul 30;19(7):e0307155. doi: 10.1371/journal.pone.0307155 (PMC11288413; doi:10.1371/journal.pone.0307155)
Supplement: S8 Table — Results of the proportion of time points and time weighted average analysis. (DOCX) [file pone.0307155.s015.docx]

| **Table S8. Proportion of time points and time weighted average** | | | | |
| --- | --- | --- | --- | --- |
|  | automated ventilation | conventional ventilation | mean difference  (95% CI) | *p* |
| Proportions of  MP > 17 J/min (%) | 31 [0–64] | 40 [14–77] | –7.51 (–20.90 to 5.88) | ns |
| Time weighted average | 15.5 [11–19] | 15 [11–21] | –0.80 (–3.16 to 1.55) | ns |
| Values are median [IQR].  Abbreviations: MP, mechanical power; CI, confidence interval.. | | | | |
