# Supplementary material for: Effect of automated versus conventional ventilation on mechanical power of ventilation—A randomized crossover clinical trial
Source: PLoS One. 2024 Jul 30;19(7):e0307155. doi: 10.1371/journal.pone.0307155 (PMC11288413; doi:10.1371/journal.pone.0307155)
Supplement: Attachment — Submitted filename: Response to reviewers def.docx [file pone.0307155.s016.docx]

To:	Dr. Samuele Ceruti

Academic Editor

PLOS ONE

From:	Dr. Laura A. Buiteman–Kruizinga

Department of Intensive Care

Reinier de Graaf Hospital

Delft, The Netherlands

&

Prof. Marcus J. Schultz

Amsterdam University Medical Centers

Amsterdam, The Netherlands

**Revised version of manuscript PONE-D-24-01411, entitled ‘Effect of Automated versus Conventional Ventilation on the Mechanical Power of Ventilation – a randomized crossover clinical trial’**

April 30, 2024

Dear editor, dear dr. Ceruti,

Thank you for providing us with the possibility to revise our manuscript entitled ‘Effect of Automated versus Conventional Ventilation on the Mechanical Power of Ventilation – a randomized crossover clinical trial’ with number PONE-D-24-01411.

The thoughtful comments by the respected reviewers were very helpful to improve our manuscript.

Of note, Laura Buiteman–Kruizinga received fees from Hamilton Medical for lecturing. Marcus Schultz was part–time employed as a team leader of Research and New Technologies at Hamilton Medical AG, Bonaduz, Switzerland, from January 2022 till January 2023. This does not alter our adherence to PLOS ONE policies on sharing data and materials. This has been reworded at your request for the Competing Interest Statement.

An itemized point–by–point response follows hereafter.

We look forward to your response, and also to the responses from the reviewers in case they get a second chance to review our manuscript.

On behalf of all co–authors, sincerely,

Dr. Laura Buiteman–Kruizinga & prof. Marcus Schultz

**Academic Editor**

1. The study is interesting and could have a good appeal; however, it is necessary to be more rigorous in the statistical and methodological part in general, not only evading what the reviewers point out, but also sticking more closely to the elements that are highlighted in the results part. It is an interesting paper, but still far from being resolved; resolving the problems highlighted by the reviewers does not yet guarantee publication of the paper.

**Thank you for your kind words. The comments of the reviewers were very helpful especially with regard to the methodological and statistical parts. We focused on staying close to the results related to mechanical power and ventilation parameters. We refer to the replies below.**

2. Please ensure that your manuscript meets PLOS ONE's style requirements, including those for file naming.

**Thank you for your guidance. We corrected all issues to meet all criteria.**

3. Thank you for stating the following in the Competing Interests section: ‘LBK received fees from Hamilton Medical for lecturing. MJS was part-time employed as a team leader of Research and New Technologies at Hamilton Medical from January 2022 till January 2023. The other authors declare no conflicts of interest.’ Please confirm that this does not alter your adherence to all PLOS ONE policies on sharing data and materials, by including the following statement: ‘This does not alter our adherence to PLOS ONE policies on sharing data and materials.’ If there are restrictions on sharing of data and/or materials, please state these. Please note that we cannot proceed with consideration of your article until this information has been declared. Please include your updated Competing Interests statement in your cover letter; we will change the online submission form on your behalf.

**Thank you for your help. We updated the Competing Interests Statement according to your suggestions, as follows:**

*‘LBK received fees from Hamilton Medical for lecturing. MJS was part-time employed as a team leader of Research and New Technologies at Hamilton Medical from January 2022 till January 2023. This does not alter our adherence to PLOS ONE policies on sharing data and materials. The other authors declare no other conflicts of interest.’*

4. In the online submission form, you indicated that ‘data are available on reasonable request including a statistical analysis plan’. All PLOS journals now require all data underlying the findings described in their manuscript to be freely available to other researchers, either 1. In a public repository, 2. Within the manuscript itself, or 3. Uploaded as supplementary information. This policy applies to all data except where public deposition would breach compliance with the protocol approved by your research ethics board. If your data cannot be made publicly available for ethical or legal reasons (e.g., public availability would compromise patient privacy), please explain your reasons on resubmission and your exemption request will be escalated for approval.

**Thank you for your suggestion about the policy, we have provided anonymized data in an online public repository figshare (**[**https://doi.org/10.6084/m9.figshare.25722435.v1**](https://doi.org/10.6084/m9.figshare.25722435.v1)**). It is under embargo for public access and will be published after the possible publication of the manuscript. A private link is created to access the files, see** [**https://figshare.com/s/5765819d443d6e91ab1a**](https://figshare.com/s/5765819d443d6e91ab1a)**.**

**Reviewer 1**

1. I advise authors, first of all, to remove abbreviations in the abstract. This ensures that the abstract is more fluid and readable. Always remaining within the limits permitted by the magazine.

**Thank you for your suggestion. We removed the abbreviations in the Abstract, which reads now as follows:**

*‘Introduction*

*Mechanical power of ventilation, a summary parameter reflecting the energy transferred from the ventilator to the respiratory system, has associations with outcomes. INTELLiVENT–Adaptive Support Ventilation is an automated ventilation mode that changes ventilator settings according to algorithms that target a low work – and force of breathing. The study aims to compare mechanical power between automated ventilation by means of INTELLiVENT–Adaptive Support Ventilation and conventional ventilation in critically ill patients.*

*Materials and methods*

*International, multicenter, randomized crossover clinical trial in patients that were expected to need invasive ventilation > 24 hours. Patients were randomly assigned to start with a 3–hour period of automated ventilation or conventional ventilation after which the alternate ventilation mode was selected. The primary outcome was mechanical power in passive and active patients; secondary outcomes included key ventilator settings and ventilatory parameters that affect mechanical power.*

*Results*

*A total of 96 patients were randomized. Median mechanical power was not different between automated and conventional ventilation (15.8 [11.5–21.0] versus 16.1 [10.9–22.6] J/min; mean difference –0.46 (95%–CI –1.61 to 0.69) J/min; P = 0.43). Subgroup analyses showed that mechanical power was lower with automated ventilation in passive patients (17.2 [12.8–23.2] versus 19.2 [14.4–24.6] J/min; mean difference –1.13 (95%–CI –1.89 to –0.37) J/min; P < 0.01), and not in active patients (14.6 [11.0–20.3] vs 14.1 [10.1–21.3] J/min; mean difference 0.82 (95%–CI –2.13 to 0.49) J/min; P = 0.22).*

*Conclusions*

*In this cohort of unselected critically ill invasively ventilated patients, automated ventilation by means of INTELLiVENT–Adaptive Support Ventilation did not reduce mechanical power. A reduction in mechanical power was only seen in passive patients.’* (Page 4; line 1 to 25, page 5; line 1 to 5)

2. Many chosen keywords are not included in the MESH database, I advise authors to balance the keywords not present with those present in order to facilitate indexing of the manuscript in case of publication.

**Thank you for your help. We limited our keywords to the following:**

*‘Intensive care; mechanical ventilation; automated ventilation; mechanical power; tidal volume; driving pressure, respiratory rate).’* (Page 5; line 10-12)

3. I advise the authors in the study intervention section to describe how the INTELLIVENT reasons and works, since otherwise it is not clear why SpO_2_ and EtCO_2_ and the other values are set on the ventilator. The authors must think about the fact that not everyone knows this type of ventilation just as not everyone knows ASV ventilation. In some parts of the manuscript, or in the introduction or better yet in a methods section, the authors must think of introducing and describing both the ASV and the INTELLIVENT which is its evolution.

**Thank you for this important comment. In the Introduction and in the Methods of the revised version of our manuscript, the principles of INTELLiVENT–ASV are more extensive explained, as follows:**

**In the Introduction:**

*‘Closed–loop, or automated ventilation modes are increasingly available for use in critically ill invasively ventilated patients [18]. INTELLiVENT–Adaptive Support Ventilation (ASV) is one automated mode that sets and adjusts VT, RR, positive end–expiratory pressure (PEEP) and the fraction of inspired oxygen (FiO2), after inserting gender and height, target ranges for the end–tidal CO2 (etCO2) and pulse oximetry (SpO2), and limits for maximum airway pressure and PEEP into the ventilator. INTELLiVENT–ASV then changes ventilator settings based on algorithms that target a low work – and force of breathing [19, 20], including settings that may affect MP [21-24].* (page 6; line 16-24)

**And Methods:**

*‘The predecessor of INTELLiVENT–ASV, in short ASV, is a ventilation mode that adapts to patients’ respiratory mechanics, providing settings as needed. It simplifies the ventilatory management process by adjusting V_T_ and RR automatically, based on the patients’ conditions. INTELLiVENT–ASV incorporates advanced algorithms and real–time monitoring to further optimize ventilation parameters and support patient breathing. Herein, PEEP and FiO_2_ are adjusted with the PEEP controller and FiO_2_ controller, and V_T_ and RR are further adjusted by a minute ventilation controller. With INTELLiVENT–ASV, the ventilation and oxygenation targets are chosen via ranges for etCO_2_ and SpO_2_ readings. The ventilator then sets and adjusts breath–by–breath the size of V_T_ in conjunction with the RR, and the level of PEEP and FiO_2_. Doctors, ventilation practitioners and nurses were extensively trained and qualified in using the ventilators used for this study, and were experienced users of the here tested automated ventilation mode INTELLiVENT–ASV.’* (Page 9; line 17-25, page 10; line 1-4)

4. Question for the authors. When clinicians set up ‘conventional’ ventilation, do they always use Hamilton ventilators or any ventilator present in the departments? Perhaps the authors should specify it, for completeness of information.

**Thank you for your question, we are very happy to explain this further. Indeed, we used the same types of ventilators during conventional ventilation, as patients crossed over from one mode to the other while receiving ventilation with the same ventilator.**

**We detailed this in the Methods, as follows:**

*‘It is worth noting that the transition between modes did not involve changing the ventilator itself, but rather only switching the ventilation mode. Patients were ventilated with the same ventilator throughout, transitioning between modes while receiving ventilation.’* (Page 11; line 3-6)

5. I advise authors to engage a little more with the existing literature. In the discussion elaborated now there is a detailed and adequate explanation of the results with different hypotheses on some results, without, however, a real comparison with the present literature. References appear every now and then but they seem almost forced. This section needs to be better renovated.

**Thank you for your comment. In the originally submitted version of the manuscript we tried to stay brief in wording about this. In the new version we elaborated on this with much more detail, as follows:**

*‘To the best of our knowledge, this is the first randomized cross–over study that determined the effects of automated ventilation by means of INTELLiVENT–ASV on MP in an unselected critically ill ICU population. Previous research has suggested benefits in terms of lower MP with INTELLiVENT–ASV, but most studies had an observational design [21-23]. Two previous studies specifically investigated the effect of INTELLiVENT–ASV compared to conventional ventilation in patients with coronavirus disease 2019 [21, 22]. These studies demonstrated a reduction in MP with INTELLiVENT–ASV, primarily driven by a lower RR. Another study assessed whether INTELLiVENT–ASV could provide lung–protective settings, resulting in lower ΔP and MP [23]. Additionally, a small RCT focused on ARDS patients [24]. Although the primary outcome was ΔP, that study found lower transpulmonary MP with INTELLiVENT–ASV due to a lower RR at the expense of a larger V_T_.*

*Similarly, in patients after cardiothoracic surgery [30], a lower MP was reported with the use of INTELLiVENT–ASV. Our findings confirm those of the previous investigations, indicating that INTELLiVENT–ASV has the potential to adjust ventilator settings in a manner that reduces MP.*

*One major finding in our study was that the automated ventilation mode lowers MP only in passive patients, and we establish a correlation between the decline in MP and a decrease in RR. In passive patients, V_T_ and RR are under full control of the automated ventilation algorithms, which target the lowest work of breathing (the ‘Otis equation’) [19] and the lowest force of breathing (the ‘Mead equation’) [20], resulting in a lower RR and a higher V_T_. This is in line with the findings of the abovementioned study in patients with severe acute hypoxemic respiratory failure due to coronavirus disease 2019 [21].*

*In actively breathing patients, MP was not affected by INTELLiVENT–ASV. In active patients, specifically those with a high respiratory drive, the tested automated ventilation mode can only increase the pressure support level in an attempt to increased V_T_, and consequently a lower RR. Of note, MP is low in active compared to passive patients. Active patients use their diaphragm promoting alveolar recruitment [31]. With this, total lung volume increases and airway pressures decline, leading to a lower MP. It should be noted, though, that there is an ongoing debate on whether MP can be calculated in active patients [32], and herein how to measure breathing effort [33] –– the equation we used for calculating MP can easily lead to an underestimation of MP in active patients [34-36].*

*If patients received conventional ventilation before randomization, the switch to the automated mode resulted in a lower MP. In patients that were already receiving automated ventilation before randomization, MP was not affected. Probably in these last patients, INTELLiVENT–ASV had already optimized the ventilator settings [37], settings that may have been taken over when starting the first study phase. The findings of the posthoc analysis in which we compared the effect of automated ventilation on MP in patients with low and relatively normal C_RS_ showed a decrease in MP only in patients with a poor lung condition. We cannot exclude that this finding was driven by the higher proportion of active patients in the group of patients with a relative normal C_RS_.*

*In clinical practice, it can be challenging to set the ventilator so that ventilation is applied with a low MP [38, 39], in particular because it is uncertain which ventilator setting to prioritize herein [40]. For example, choosing a lower V_T_ may cause a reduction in MP [2], but if a compensatory higher RR is needed, any effect on MP could be nullified, or worse. Indeed, an increase in RR will increase MP [34]. Our study shows how an automated mode of ventilation that targets a low work – and force of breathing automatically sets ventilator settings so that MP is reduced. No manual interventions by ICU nurses or doctors were needed.*

*Thus far, lung–protective ventilation has focused on the use of a correct, i.e., low V_T_ [12], and less on the potential injurious effects of the RR [41]. One salient finding in our study was that with the tested automated ventilation mode V_T_ actually increases, and RR decreases. Recent studies show that ventilation strategies with use of low V_T_ and higher RR may only be beneficial in patients with low C_RS_ [17, 42], while it results in harm in patients with a relative normal C_RS_. Interestingly, in patients with a low C_RS_ the automated ventilation mode chooses a lower RR and a higher V_T_, resulting in less MP. Future studies are needed to understand whether this reduction in MP [32], achieved by a reduction in RR and an increase in V_T_ translates in better outcomes [43], such as duration of ventilation and maybe even mortality [44].’* (Page 20-21 line 17-25)

**Reviewer 2**

1. Please, provide the data and the code used to obtain the results discussed in paper. This ensures the reproducibility of the results. It would be a plus with no doubts.

**Thank you for your suggestion, we totally agree on the importance of data sharing and we support transparency. We have provided anonymized data as well as the codes used anonymized data in the online public repository figshare (**[**https://doi.org/10.6084/m9.figshare.25722435.v1**](https://doi.org/10.6084/m9.figshare.25722435.v1)**). It is under embargo for public access and will be published after the possible publication of the manuscript. A private link is created to access the files, see** [**https://figshare.com/s/5765819d443d6e91ab1a**](https://figshare.com/s/5765819d443d6e91ab1a)**.**

2. The employed statistical methods are sound. I really appreciate the use of mixed models. Nevertheless, there are several aspects which should be carefully checked. Firstly, the Gauss-Markov assumptions must be met; please, provide an extensive residual analysis confirming that the linear model is suitable for the data at hand and, if skewness, outliers, etc., arise, please modify the model accordingly. Secondly, the data are not longitudinal but also multilevel, as multiple centers are considered: please, add random effects at the center level too. As far I can see, the quantile (median) regression with random effects could be a practical and useful alternative, as long as the residuals do not look Gaussian-distributed.

**Thank you for your suggestions. We have checked that the models meet the Gauss–Markov assumptions. We added this to the Supplement. We also added the centers as random effect, and results were adjusted in the manuscript accordingly, but it did not change anything substantial. We argue against performing a median regression as this was not preplanned and will not change the message of the manuscript.**

**We adjusted the text in the Methods:**

*‘For the primary analysis, we first compared MP with automated ventilation to conventional ventilation in all patients, and then in two subgroups, i.e., patients that were passive and patients that were actively breathing at all time points. Herein, MP between ventilation modes was compared using a mixed–effect generalized linear model with Gaussian distribution, including time of measurements as a continuous variable, treatment group (automated or conventional), period (1 or 2) and sequence (automated to conventional, or conventional to automated) as fixed effects, and patients and centers as random effect to account for repeated measurements. The models were checked to meet the Gauss-Markov assumptions (S1–S3 Fig).’* (Page 12; line 14-22)

**and added the following to the Supplement:**

‘*The Gauss–Markov assumptions for residuals in a mixed–effect generalized linear model confirms that the data is suitable for the model. They include linearity, independence, homoscedasticity, normality and collinearity of residuals. We visually inspected plots to validate these assumptions, ensuring the model's validity and robustness.*’ (Supplement S1-S3 Fig)

***eFigure S1****. Figures of the residual analysis, of mixed-effect generalized linear model for the primary endpoint, including all patients;* ***eFigure S2****. Figures of the residual analysis, of the mixed-effect generalized linear model including only passive patients;* ***eFigure S3****; Figures of the residual analysis, of the mixed-effect generalized linear model including only active patients*

3. As the number of predictors and confounders is high, please provide guidance towards variables selection in the mixed effect regression framework. Please, bear in mind that it is of fundamental importance to keep confounders (mainly demographic variables) in the model to ensure that differences in the outcome do not arise because of omitted variables and heterogeneity in general.

**Thank you for this suggestion. As this is a crossover study, meaning that every patient served as his or her own control, it is not necessary to include demographic variables. However, including other variables are important to account for the crossover design. We included time of measurements (to account for time of repeated measurement) treatment group (automated or conventional ventilation), study phase (1 or 2) and sequence (automated to conventional, or conventional to automated). This is described in the statistical analysis plan in the Methods:**

*‘Herein, MP between ventilation modes was compared using a mixed–effect generalized linear model with Gaussian distribution, including time of measurements as a continuous variable, treatment group (automated or conventional), period (1 or 2) and sequence (automated to conventional, or conventional to automated) as fixed effects, and patients and centers as random effect to account for repeated measurements.’* (Page 12; line 16-21)

4. About missingness, the authors state ‘We made no assumption for missing data’. I guess that a missing completely at random mechanism is considered, am I right? I strongly suggest to investigate the role of missingness, as missing not at random may strongly bias the results. Indeed, dating back to the taxonomy introduced by Rubin, it is well known that ignoring the role played by missing values may strongly affect statistical inference. I suggest to look at pattern mixture approaches, as they are rather simple to be implemented.

**Thank you for this suggestion. Missing data was very minor, less than 0.06% and happened randomly. We decided to make no assumption for missing data, because we do not analyze patient–centered, clinical outcomes, but physiological outcomes instead. In addition, the linear mixed models we used (lme4 package in R) account for missing data. We agree the importance of knowing the missing data in a dataset. We have added the missing data to the Supplement and clarified the amount of missing data in the manuscript.**

*‘Missing data occurred completely at random. The proportion of missing data was very low (< 0.06%) and we made no assumptions for missing data.’* (Page 13; line 21-23)

5. Even for simple t-tests, please check the underlying assumptions and provide evidence (in a supplement) that inference is valid.

**We did not perform t-tests?**
